# Total dosage of gardenia fruit used by patients with mesenteric phlebosclerosis

**DOI:** 10.1186/s12906-016-1182-1

**Published:** 2016-07-11

**Authors:** Yutaka Nagata, Tetsuo Watanabe, Kazuhiko Nagasaka, Masaaki Yamada, Masafumi Murai, Sunao Takeuchi, Mai Murase, Toshinori Yazaki, Takayuki Murase, Kenichi Komatsu, Machiko Kaizuka, Mika Sano, Koji Asano, Chikao Ando, Norihide Taniuchi

**Affiliations:** Department of Oriental Traditional Medical Center, Suwa Central Hospital, 4300 Tamagawa, Chino, Nagano 391-8503 Japan; River Side Clinic, Chino, Nagano Japan; Department of Japanese Oriental Medicine and Rheumatology, Toyama Prefectural Central Hospital, Toyama, Toyama Japan; Department of Epidemiology and Health Policy, Graduate School of Medicine and Pharmaceutical Sciences, University of Toyama, Toyama, Toyama Japan; Hokudaimae Clinic, Hokkaido Kampo Medical Center, Sapporo, Hokkaido Japan; Shinsapporo Keiaikai Hospital, Sapporo, Hokkaido Japan; Department of Internal medicine, Suwa Central Hospital, Chino, Nagano Japan; Department of Surgery, Suwa Central Hospital, Chino, Nagano Japan; Department of Radiology, Suwa Central Hospital, Chino, Nagano Japan; Department of Pathology, Suwa Central Hospital, Chino, Nagano Japan

**Keywords:** Gardenia fruit, Herbal medicine, Kampo, Mesenteric phlebosclerosis, Sanshishi

## Abstract

**Background:**

Mesenteric phlebosclerosis (MP) is a disease characterized by fibrotic change or calcification of the mesenteric vein. Recently, there has been an increase in case reports of MP related to herbal medicine usage. Long-term intake of gardenia fruit (GF) is suspected as a possible cause. However, many GF users do not develop this disease and the association between GF and MP remains unclear. In this study, we investigated for the first time the dosage of GF used by patients with and without MP.

**Methods:**

We used a medical chart review study design to assess the association between GF and MP. We reviewed patients with a history of intake of herbal medicines containing GF. Among these patients, we selected patients who were examined by colonoscopy and abdominal plain computed tomography (CT). We investigated the findings of colonoscopy, CT scan and histological examination. We assessed the total dosages of GF alongside the duration of ambulatory visit, the administration period of herbal medicine containing GF and pre-existing disease in order to compare MP cases and non-MP patients.

**Results:**

Ten MP cases and 42 non-MP patients were analyzed. We summarized clinical findings of MP cases. All MP cases used more GF than non-MP patients and were administered more than approximately 5,000 grams of GF in cumulative dosage.

**Conclusions:**

This study indicated that excessive intake of GF contributes to and/or accelerates the development of MP suggesting that long-term usage of GF in excessive amounts increases the risk of MP.

## Background

Mesenteric phlebosclerosis (MP) is a relatively new disease entity [1] and is also known as phlebosclerotic colitis [2–15]. Recently, it has been termed ‘idiopathic’ mesenteric phlebosclerosis [16–22], because its etiology remains unclear. In typical MP cases, abdominal X-rays and computed tomography (CT) scans demonstrate spotted or linear calcification around the right hemicolon [2, 8]. The bronze coloration of colonic membrane is characteristic findings of this disease. In advanced MP cases, edema, ulceration, rigidity and stenosis on endoscopic examination are also observed [1]. Several cases have been reported as chronic ischemic colitis [23, 24] and chronic ischemic lesions [25–28].

Previously published cases indicated an association with several conditions such as portal hypertension as a result of liver dysfunction [3, 4, 13], blood coagulation disorders [24], CREST syndrome [29], dialysis [15], vasculitis [6], diabetes mellitus [30, 31], hyperlipidemia [30] and hypertension [31] related to MP. Recently, the number of case reports of MP with a history of intake of herbal medicine has increased [9, 16–18, 32–34]. In particular, gardenia fruit (GF), ‘Sanshishi’ in Japanese, is attracting attention as a possible cause [33, 34]. Almost all of the reported herbal medicine-related MP cases took GF over a long period of time [33, 34]. As such, some are of the opinion that it is desirable to avoid long-term usage of medicines containing GF [9, 33].

It is also the case, however, that there were several patients that had taken GF for a longer period of time than MP cases in our experience [34]. There has not been sufficient data to assess this discrepancy. In this study, we first reviewed the dosages of GF in patients with or without MP.

The aim of this study was to review the dosage of GF used by patients with and without MP, and to resolve above-mentioned discrepant issue.

## Methods

### Patients

We reviewed ambulatory patients with a history of Kampo treatment from December 1, 2013 to May 25, 2015. Among the patients that reported usage of herbal medicines containing GF, we selected patients who were examined by colonoscopy and abdominal plain CT. We reviewed the findings of these clinical examinations performed from June 4, 2010 to May 25, 2015. A series of examinations were performed at the following facilities: Suwa Central Hospital, River Side Clinic and Shinsapporo Keiaikai Hospital.

### Herbal medicines containing gardenia fruit

We used two dosage forms of Kampo preparations, namely extract preparations and traditional decocted herbal medicines. Medical-grade extract preparations of Kampo formulas containing GF as one of the ingredients used in Japan are listed in Table 1.

**Table 1:** Medical-grade extract preparations of Kampo formulas containing gardenia fruit

Kampo formula	GF (g/day)	Corporations manufacturing and marketing
Bofutsushosan	1.2 g	Hon, JPS, Kot, Kra, Mat, Osu, San, Tai, Toy, Tsu
Gorinsan	2.0 g	Toy, Tsu
Inchinkoto	3.0 g	Kot, Kra, Osu, Toy, Tsu
	2.0 g	Tei
Kamikihito	2.0 g	Kra, Osu, Tai, Toy, Tsu.
Kamisyoyosan	2.0 g	Hon, Jun, JPS, Kot, Kra, Mat, Osu, San, Tai, Tei, Toy, Tsu
Keigairengyoto	1.5 g	Osu, Tai, Tei, Tsu
Orengedokuto	2.0 g	Hon, Jun, JPS, Kot, Kra, Osu, Sak, San, Tai, Tei, Toa, Toy, Tsu
Ryutansyakanto	1.5 g	Kot, Tai
	1.0 g	San, Tsu
Saikoseikanto	1.5 g	Kot, Tei, Tsu
Seihaito	2.0 g	Tsu
Seijobofuto	2.5 g	Tsu, Osu
Shin’iseihaito	3.0 g	Kot, Tsu, Osu
	1.5 g	Kra
Shishihakuhito	3.0 g	Kot
Unseiin	1.5 g	Kra, Osu, Tei
	2.0 g	Toy, Tsu, Hon, Jun, Kot

*GF* gardenia fruit, *Hon* Honzo, *Jun* Junko, *Kot* Kotaro, *Kra* Kracie, *Mat* Matsuura, *Osu* Osugi, *Sak* Sakamoto, *San* Sanwa, *Tai* Taikodo, *Tei* Teikoku, *Toa* Toayakuhin, *Toy* Toyoyakko, *Tsu* Tsumura

### Colonoscopy and CT scan

Upon receipt of patient consent, colonoscopy and CT scan were performed to screen for MP and other diseases. We considered the diagnosis of MP from the findings of these clinical examinations and histological inspection [34]. Thickening of the affected colonic wall with calcification and associated calcification of the mesenteric vein and its tributaries on CT scan confirmed the presence of MP [2, 7]. We regarded the following findings as valuable changes at the early stage: slight calcification of the right hemicolon, increase in CT values in surrounding adipose tissue [4] and mild dilatation of the ileocecal vein.

### Histological inspection

A diagnosis of MP was confirmed with histological inspection. Fibrotic deposition observed in the pericapillary region of the lamina propria [1] was the diagnostic criteria used in our hospital in this study. The specimens for examination were collected by colonoscopic biopsy. We used an excision sample of a colectomy in a patient undergoing an emergency operation. The biopsy was performed in a case in which the color change of the colonic membrane was suspected. The biopsy specimens were collected from a location with a mild blue coloration or a characteristic bronze color change. We evaluated the degree of fibrillization and associated reduction of ductal cell density in the lamina propria (Additional file 1: Table S1) [34].

### Medication history and total dosages of gardenia fruit

We reviewed the medication history of various kinds of herbal medicine containing GF. Moreover, we investigated the administration period of these medicines and the duration of ambulatory visit from clinical records. We excluded patients from this study who obtained GF-containing drugs from other facility. All patients started taking of GF at our facilities.

This allowed for investigation of duration from the beginning to the end of use for herbal medicines containing GF. We reviewed clinical records that recorded from January 23, 1997 to May 25, 2015.

We also calculated cumulative GF dosage, which is presented in Table 1. Various dosages of GF are consumed depending on the manufacturing of each drug. Mixtures of GF and other crude drugs are extracted with water for manufacturing a freeze-dried extract preparation. Concretely, only with the Bofutsushosan extract does, the dosage of GF vary from 0.4 g in a single administration (2.5 g/one packet) to 1.2 g in a daily dose (7.5 g/3 packets). In addition, we used traditional medicines, decocted with a mixture of crude drugs and water. The quantities of one-time dosages of GF in decocted traditional medicines were 1–3 g. We calculated the cumulative dosage of GF administered to each patient from such data.

### Pre-existing diseases

We also investigated pre-existing diseases. The diagnostic criteria for pre-existing diseases were as follows: [elevated triglyceride (TG)]: TG ≥ 150 mg/dL or taking therapeutic drug for a hypertriglyceridemia, [elevated low-density lipoprotein cholesterol (LDL-c)]: LDL-c ≥ 140 mg/dL or taking a therapeutic drug for hypercholesterolemia, [hypertension (HT)]: systolic blood pressure ≥ 130 mmHg and/or diastolic blood pressure ≥ 85 mmHg or taking an antihypertensive drug, and [diabetes mellitus (DM)]: fasting plasma glucose ≥ 110 mg/dL or HbA1c (NGSP) ≥ 6.5 % [35] or taking an oral hypoglycemic agent. These criteria were in agreement with criteria proposed by the Examination Committee for Metabolic Syndrome in Japan [36].

### Comparison between MP cases and non-MP patients

We assessed the duration of ambulatory visit, the administration period of herbal medicine containing GF, cumulative GF dosage and pre-existing disease in order to compare MP cases and non-MP patients.

### Statistical analysis

Statistical analysis was performed with JMP 9 (SAS Institute Japan, Tokyo). Data were expressed as mean ± S.D.. Either a Student *t* test, Fisher’s exact test or Mann–Whitney *U* test were used for statistical analysis of patient characteristics. A value of *P* < 0.05 was considered statistically significant.

## Results

### Reviewed patients

We reviewed 1,927 patients with a history of Kampo treatment from December 1, 2013 to May 25, 2015. Two-hundred and forty-nine patients took herbal medicines containing GF.

### MP cases of enrolled patients

This study enrolled 52 patients that had used GF-containing herbal medicines and received colonoscopy and/or CT scan. We found 10 MP cases in total at three institutions (Table 2); 5 were male, and 5 were female. The mean age at the time of diagnosis of MP cases was 66.1 years (range: 49 to 76 years old). All MP cases were diagnosed by histological inspection with colonic biopsy specimen or excision sample from an operation. One case (No. 3) developed into acute abdomen and was given a diagnosis of MP during emergency surgery. Two cases (No. 1, 10) were accidently found in CT. The other two cases were accidentally (No. 6, 8) pointed out in colonoscopy. The other five cases were diagnosed by a screening examination for MP as we actively searched for undiagnosed cases of MP actively with colonoscopy and CT scan.

**Table 2:** Characteristics and clinical findings of 10 MP cases

No. Age Sex	Chief complaint/MP symptom	Kampo formulas containing GF/Clinical findings	GF intake cumulative dosage Administration period	Pre-existing disease
1. 76 M	*Chief complaint* Hypertension	Orengedokuto	15,792 g 14.1 years	DM, HT, LDL-c
	*MP symptom* none	*Clinical findings (location)* *Colonoscopy:* Bronze coloration (C to T) Redness and Erosion (C to A) *Histology:* Moderate fibrillization *CT scan:* Linear calcification (C to T) Wall thickening (C to A)		
2. 76 M	*Chief complaint* Hypertension	*Orengedokuto,* Orengedokuto, Ryutansyakanto	14,683 g 15.9 years	HT, LDL-c
	*MP symptom* none	*Clinical findings (location)* *Colonoscopy:* Bronze coloration (C to T) *Histology:* Severe fibrillization *CT scan:* Linear calcification (C to T) Wall thickening (C to A)		
3. 70 M	*Chief complaint* Hypertension	Orengedokuto, Kamisyoyosan, Ryutansyakanto, Seihaito	8,119 g 11.9 years	DM, HT, TG
	*MP symptom* Ileus (Colectomy)	*Clinical findings (location)* *Histology:* Fibrillization of the colonic mucosa and the mesenteric vein in excision sample *CT scan:* Linear calcification (C to T) Wall thickening (C to A)		
4. 49 F	*Chief complaint* Skin itching	Unseiin, Kamisyoyosan, Keigairengyoto, Orengedokuto	7,972 g 12.2 years	LDL-c
	*MP symptom* none	*Clinical findings (location)* *Colonoscopy:* Mild blue coloration (C to A) Decreased transparency of blood vessels (C to A) *Histology:* Mild fibrillization *CT scan:* Slight calcification, CT value increase, Mild vasodilation (the ileocecal vein)		
5. 75 M	*Chief complaint* Cutaneous pruritus	*Unseiin*	6,579 g 6.8 years	HT
	*MP symptom* Constipation Abdominal pain	*Clinical findings (location)* *Colonoscopy:* Dark blue coloration (C to T) Redness and Erosion (C to A) *Histology:* Severe fibrillization *CT scan:* Linear calcification (C to T) Wall thickening (C to T)		
6. 58 F	*Chief complaint* Dermatitis and Obesity	Bofutsusyosan, Unseiin, Keigairengyoto, Orengedokuto	6,499 g 13.5 years	LDL-c
	*MP symptom* none	*Clinical findings (location)* *Colonoscopy:* Edematous membrane and Bronze coloration (C to A) *Histology:* Concentric fibrillization surrounding capillary vessel		
7. 68 M	*Chief complaint* Cutaneous pruritus	*Unseiin*	6,372 g 12.2 years	DM, HT, LDL-c, TG
	*MP symptom* none	*Clinical findings (location)* *Colonoscopy:* Bronze coloration (C to T) *Histology:* Severe fibrillization *CT scan:* Linear calcification (the ileocolic vein)		
8. 58 F	*Chief complaint* Hot flush	Kamisyoyosan, Kamikihito, Orengedokuto, Shin’iseihaito	5,588 g 4.0 years	LDL-c, TG
	*MP symptom* none	*Clinical findings (location)* *Colonoscopy:* Bronze coloration (C to T) *Histology:* Mild - Moderate fibrillization *CT scan:* Slight calcification and CT value increase (C to A)		
9. 64 F	*Chief complaint* Flushed face, Feeling of cold	*Kamisyoyosan*	5,408 g 11.4 years	HT, LDL-c, TG
	*MP symptom* none	*Clinical findings (location)* *Colonoscopy:* Mild blue coloration (C to T) *Histology:* Mild - Moderate fibrillization *CT scan:* Linear calcification (C to A)		
10. 67 F	*Chief complaint* Hepatitis	Orengedokuto	5,379 g 9.4 years	Liver disease
	*MP symptom* none	*Clinical findings (location)* *Colonoscopy:* Mild blue coloration (C to T) *Histology:* Mild - Moderate fibrillization *CT scan:* Linear calcification (C to T) Wall thickening (C to A)		

*CT* computed tomography, *GF* gardenia fruit, *MP* mesenteric phlebosclerosis, *DM* diabetes mellitus, *HT* hypertension, *LDL-c* elevated low-density lipoprotein cholesterol, *TG* elevated triglyceride, *C* the caecum, *A* the ascending colon, *T* the transverse colon, *Oblique type medicines*, traditional decocted herbal medicines

We scrutinized the endoscopic findings of 9 MP cases receiving colonoscopy. Typical bronze coloration was observed in 5 of 9 MP cases. Four cases remained a mild or dark blue coloration on colonoscopy.

**Fig. 1:**
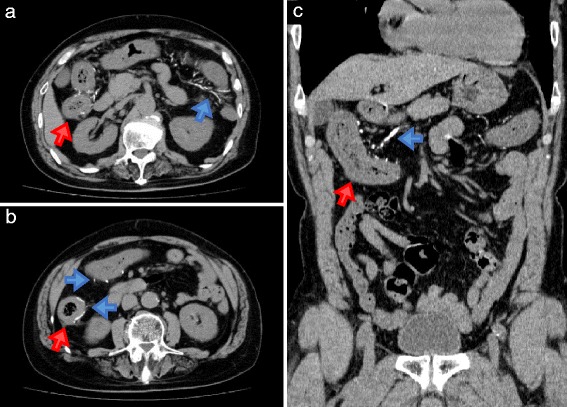
Typical findings of CT scan in MP cases (No. 2 in Table 2). Typical calcification and wall thickening in the transverse colon (**a**) and the ascending colon (**b**). Linear calcification in the transverse colon in coronal section (**c**). The blue colored arrows indicate the typical calcification. The red colored arrows indicate the thickening in the colon. CT, computed tomography; MP, mesenteric phlebosclerosis

Concerning the findings of CT scan, thickening of the affected colonic wall with calcification was observed in 5 of 9 MP cases who had a CT scan. Linear calcification of the mesenteric vein and its tributaries on CT scan was observed in 7 of 9 cases. Typical CT images were observed with a MP case (No. 2 of Table 2) (Fig. 1). Two cases continued to have slight calcification. Mild vasodilation of the ileocecal vein was observed in 1 of 9 cases.

**Fig. 2:**
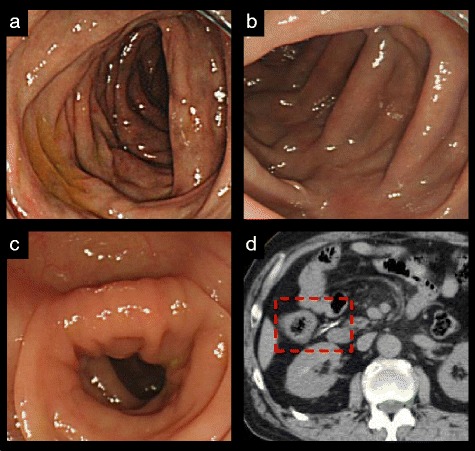
Clinical findings of colonoscopy and CT scan in MP cases (No. 1 in Table 2). Bronze coloration and redness in the ascending colon (**a**), bronze coloration in the hepatic flexure (**b**) and normal membrane in the descending colon (**c**) on colonoscopy. Linear calcification and wall thickening in the ascending colon (**d**) on CT scan. CT, computed tomography; MP, mesenteric phlebosclerosis

**Fig. 3:**
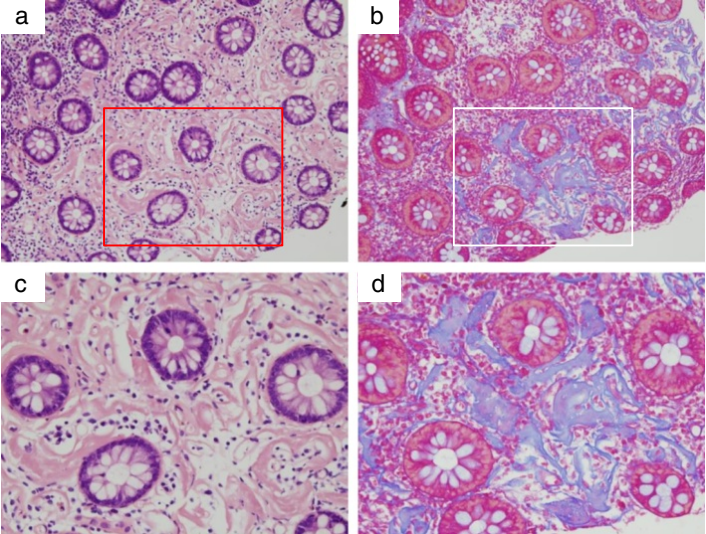
Histological findings in MP Case (No.1 in Table 2). Moderate pericapillary concentric fibrillization was noted in the specimens collected at the ascending colon. The fibrillization was associated with a reduction of ductal cell density in part of specimen. [Haematoxylin and Eosin stain: **a** x10 : **c** x20]. [Azan stain: **b** x10, **d** x20]. MP, mesenteric phlebosclerosis

The maximum dose of the MP cases was 15,792 g (No. 1 of Table 2). Colonoscopy showed typical bronze mucosa extending from the cecum to the transverse colon. Moreover, redness and erosion of the colonic membrane were observed in the cecum and the ascending colon (Fig. 2a, b). There was no color change in the descending colon (Fig. 2c). Severe wall thickening and typical linear calcification were observed on CT scan (Fig. 2d). The histological findings of moderate fibrotic lesions in pericapillary region of the lamina propria were observed (Fig. 3). Fibrillization was associated with a reduction of ductal cell density in part of the specimen.

### Pre-existing disease

All MP cases had pre-existing disease (Table 2). Six MP cases had hypertension, and LDL-c was elevated in seven MP cases. There was no significant difference in each factor between MP cases and non-MP patients in this study (Table 3). Of 10 cases with MP, only one female case had liver disease. Although her dosage of GF was minimal in MP cases, typical linear calcification and wall thickening was observed.

**Table 3:** Comparison of non-MP patients with MP cases

	non-MP (*n* = 42)	MP (*n* = 10)	*P* value
Age (year)	58.2 ± 14.8	66.1 ± 9.1	.1113^a^
	58.5 (16–86)	67.5 (49–77)	
Sex (male/female)	11/31	5/5	.2509^b^
BMI (kg/m^2^)	22.5 ± 3.8	23.0 ± 2.3	.7024^a^
	22.5 (16.0–31.4)	22.8 (19.5–27.7)	
DM (yes/no)	5/37	3/7	.1710^b^
HT (yes/no)	14/28	6/4	.1562^b^
LDL-c (yes/no)	19/23	7/3	.2913^b^
TG (yes/no)	8/34	4/6	.2125^b^
Liver disease (yes/no)	0/42	1/9	.1923^b^
Herbal medicine treatment			
Ambulatory visit (years)	6.8 ± 4.3	13.9 ± 3.0	.0001^c^
	6.3 (1.5–17.9)	15.1 (6.5–16.3)	
GF administration period (years)	4.2 ± 3.7	11.1 ± 3.5	.0001^c^
	3.1 (0.2–14.8)	12.1 (1.5–15.9)	
Cumulative dosage of GF (g)	1319.7 ± 1077.7	8239.1 ± 3817.4	< .0001^c^
	997.5 (54.0–4542.0)	6539.0 (5379.0–15792.0)	

Data are expressed as mean ± S.D. and median (minimum - maximum)

*MP* mesenteric phlebosclerosis, *BMI* body mass index, *DM* diabetes mellitus, *HT* hypertension, *LDL-c* elevated low-density lipoprotein cholesterol, *TG* elevated triglyceride, *GF* gardenia fruit

^a^Comparison between non-MP group and MP group by Student’s *t* test

^b^Comparison between non-MP group and MP group by Fisher’s exact test

^c^Comparison between non-MP group and MP group by Mann-Whitney *U*-test

### GF administration period

**Fig. 4:**
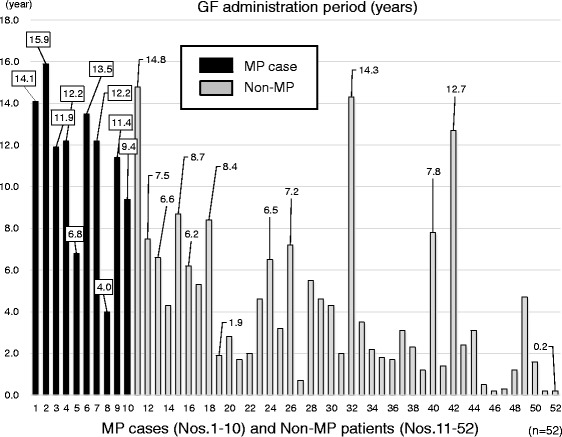
GF administration period (years) in examined cases with or without MP (n = 52). There were several non-MP patients who exceeded MP cases for duration of administration period of GF. They did not develop MP in spite of their long-term GF administration. GF, gardenia fruit; MP, mesenteric phlebosclerosis

The average administration period was 11.1 years (range: 4.0 to 15.9 years) in MP cases (Table 3). We also investigated the relation of the administration period of GF for MP cases and non-MP patients. Regarding this point, GF was administered in several non-MP patients longer than the shortest administration period of MP cases (Fig. 4). However, there were significant differences in the duration of ambulatory visit and GF administration period between MP cases and non-MP patients (Table 3).

### Cumulative dosage of gardenia fruit

**Fig. 5:**
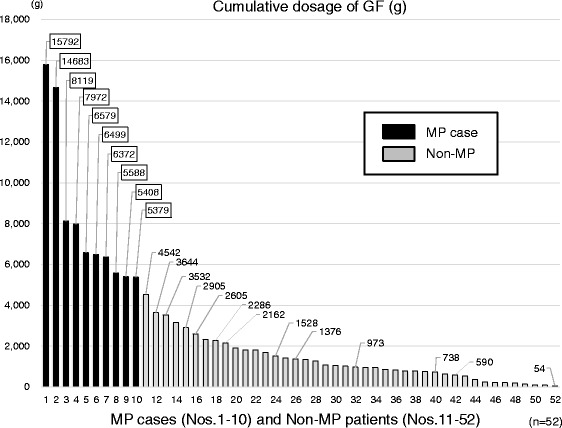
Cumulative GF dosage (g) in examined cases with or without MP (*n* = 52). The total dosages of GF consumed by MP cases were larger than those in other 42 patients. The total amount of 5,379 - 15,792 g of GF was used in MP cases. The maximum dose of the non-MP patients was 4,542 g. GF, gardenia fruit; MP, mesenteric phlebosclerosis

We investigated the cumulative dosages of GF in all 52 patients, including the 10 MP cases (Fig. 5). The ten cases with a definitive diagnosis of MP had all used more GF than non-MP patients. There was a remarkably significant difference between MP cases and non-MP patients in cumulative GF dose (Table 3).

The minimum dosage of GF was 5,379 g in MP cases. The maximum dose of GF administered to non-MP patients was 4,542 g. There was a constant distinction in GF dosage for MP diagnosed cases and non-diagnosed patients (Fig. 5).

### Relationship between GF administration period and GF dose

**Fig. 6:**
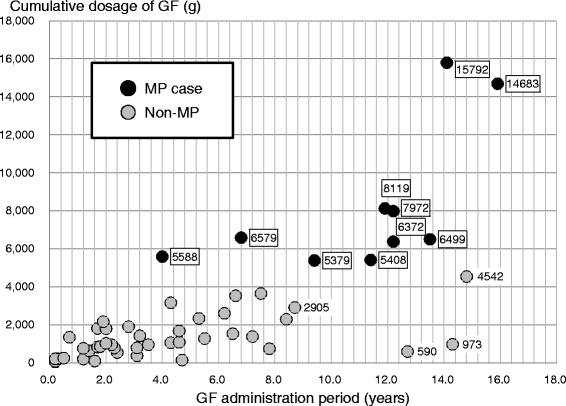
Relation between GF administration period and cumulative dosages of GF (*n* = 52). There were several patients that had taken GF for a longer period of time than MP cases. All MP cases used more GF than non-MP patients. GF, gardenia fruit; MP, mesenteric phlebosclerosis

We presented a relation between GF administration period and cumulative dosages of GF in each patient in all 52 subjects in Fig. 6. Although there were several non-MP patients that had taken GF for a longer period of time than MP cases, all non-MP patients used less GF than all MP cases.

## Discussion

In this study, we reviewed 10 MP cases and 42 non-MP patients. All MP cases had used more GF than non-MP patients. Our results suggested that excessive intake of GF contributes to and/or accelerates the development of MP.

The essential pathology of “phlebosclerosis” has been described as a fibrous degeneration of the intima and/or media and adventitia with or without calcification [37]. Some pathogenic and etiological factors such as inflammation, thrombosis, mechanical stress and calcium deposition are mentioned, however, the role of these factors have not been established.

Although the actual cause and pathogenesis of MP remains largely unclear, GF has attracted attention as a possible cause of MP [9, 33]. Published reports provided three viewpoints regarding the suspected association between GF and MP: (1) most herbal medicine-related MP cases took GF for a long time, (2) most MP cases were reported from the Asia region where GF is commonly used, and (3) a favorite site of MP is the right hemicolon where the constituents of GF are largely metabolized. Our results appear to support and rationalize these suggestions.

Firstly, GF has been reported as a common ingredient used in MP cases with a history of herbal treatment [33]. Similarly, all MP cases at our facilities had used GF and there was no other common ingredient apart from GF in these cases. The long period, spanning several years or over a decade, suggested that such a large quantity of GF can explain the long-term intake of GF in MP cases.

In previous research, the average length of herbal treatment leading to development of MP was reported as 13.6 years in the largest-scale study [38]. In this study, the average length of GF administration period was 11.1 years in MP cases (Table 3). We paid particular attention to initial changes such as a thickened colon and elevated CT value on CT scans, and slight color change on colonoscopy. This might have allowed us to detect MP cases earlier than other facilities.

Secondly, an association between GF and MP can be explained by regional/cultural factors. It is characteristic that almost all reported cases of MP were in individuals from Asian countries such as Japan [1, 2, 4, 5, 7, 8, 16, 17], Taiwan including Taiwanese residents in Canada [10, 12, 14, 18, 20, 39], Hong Kong [11, 40], Korea [13, 19, 41] and China including a Chinese resident in the USA [15, 21, 22, 32]. GF has long been used in eastern Asia as a crude drug or coloring agent. GF is a one of the most useful herbal medicines applied in Kampo treatment. Herbal medicines containing GF are often necessary to improve a patient's quality of life. They are useful for the relief of various symptoms (Additional file 2: Table S2) and have been used in the treatment of several pathological conditions [42–50]. In fact, symptoms of our MP cases were ameliorated by administration of Kampo formulas containing GF. Therefore, patients wanted to receive these drugs. As such, Asian people have a high chance of taking more GF than residents of other regions.

Finally, a favorite site of MP is common to the section where the constituents of GF are largely decomposed by intestinal bacteria. Previous reports supposed that certain toxic biochemical agents or water-soluble irritants absorbed from the ascending colon cause chronic damage [16, 18]. We also consider that sustained stimulation with a suitable dose of GF could form a MP lesion. The constituents of herbal medicine are orally administered and brought into contact with enteric bacterium. They are metabolized to be bioactive, ineffective, or toxic compounds before they are absorbed from the gastrointestinal tract [51]. It is reported that intestinal bacteria play an important role in the intestinal metabolism of GF-components [52, 53]. Because the distal ileum and colon have bacteria in large quantities, the main area affected by the metabolites passing through it might be at the right side colon [54].

The metabolism of the main constituents of GF has been previously studied [53, 55–57]. Metabolites from GF include eight major bioactive constituents, including geniposidic acid, chlorogenic acid, genipin-1-β-gentiobioside, geniposide, genipin, rutin, crocin-1 and crocin-2 [57]. Among these constituents, geniposide has been suggested as a possible cause of MP [33]. Geniposide-induced hepatotoxicity has been reported previously [58, 59]. The hepatotoxicity of high doses of geniposide has also been reported to be linked to oxidative stress [59]. Geniposide, a main iridoid glucoside component of GF, is converted to aglycone, genipin [55, 56]. When cross-linked with amino acid, genipin can be observed as having a blue coloration [60]. This might relate to the blue-colored mucosa observed in MP. Collectively, the ingredients of GF seem to have some kind of influence, but it is still unknown how they specifically act on MP.

Until recently, it was not known that GF had the potential to be harmful. It is now important, however, to consider the possible development of MP. When MP progresses without clinical checkup, mesenteric circulatory failure may induce severe constipation, ileus, bowel dysfunction and ischemic colitis. Moreover, total or partial colectomy has often been performed for such severe cases [3, 4, 6, 26, 28]. As such, due to various concerns in the literature, calls for awareness and caution associated with GF and MP have been raised. We are also cautious, however, about the possibility that excessive concern keeps away a patient from effective treatment. Currently, without sufficient data, special attention should be given to any lengthy period of GF use that spans over years.

Looking more carefully at longer periods of use, we confirmed in this study that several non-MP patients had taken GF for a longer period of time than MP cases. These non-MP patients were administered GF intermittently and/or at a low daily dose of GF. Therefore, if the cumulative dose of GF is kept to low levels, MP occurrence or development is likely to be avoided.

MP is sometimes missed or misdiagnosed due to a lack of typical findings. However, we were able to detect early MP with histological inspection in a colonic biopsy specimen. If there are findings such as a mild blue coloration of the colonic membrane, a biopsy should be performed. However, it is difficult to diagnose MP in this way since most patients do not wish to undergo a colonoscopy. In such cases, the overall cumulative dose of GF might provide a criterion for determining whether or not the patient has MP. Even if these examinations are not performed, it is recommended that any patients with excessive intake of GF should refrain from further use.

The most notable result of this study is an association between GF intake and the emergence of MP. Our results suggest that an excessive intake of GF, approximately 5,000 g in this study, contributes to and/or accelerates the development of MP. The threshold may increase or decrease with the contribution of several other conditions. It is also necessary to consider etiologies of MP other than GF. We confirmed all MP cases had pre-existing disease, but there was no significant difference in each lifestyle disease between MP cases and non-MP patients in this pilot study. However, we had an impression that they were numerically different. The non-significant results in this study could be because of the small sample size, particularly in the MP group (n = 10). The correlation between pre-existing disease and onset of MP can be validated in additional investigation with larger patient population. Further verification of the relationship with other risk factors is needed. Although our study suggested an association between MP development and cumulative GF dose, we did not reach an identification of the causal ingredient in GF. Thus, identifying the actual toxic agent is our next task.

This study is the first report about the total GF cumulative dose with or without MP in patients confirmed to have taken GF. Specifically, the cumulative dosage of GF seems to be responsible for developing MP rather than the duration of its administration.

### Limitations

We excluded patients who were already taking GF at another medical facility prior to coming to our facilities from the study. We also excluded patients who took over-the-counter herbal preparations containing GF. If this was not recorded in the medical charts, it could be possible that some patients had obtained GF containing medical-grade preparations from another medical facility or GF-containing over-the-counter preparations from a pharmacy. If such cases exist, then it is possible that the threshold for cumulative GF dosage may be higher.

## Conclusion

In this study, all MP cases used more GF than non-MP patients. This result indicated that excessive intake of GF contributes to and/or accelerates the development of MP, suggesting that long-term usage of GF in excessive amounts increases the risk of MP.

## Abbreviations

CT, computed tomography; DM, diabetes mellitus; GF, gardenia fruit; HT, hypertension; LDL-c, low-density lipoprotein cholesterol; MP, Mesenteric phlebosclerosis; TG, triglyceride
